# A *p*-orbital honeycomb-Kagome lattice realized in a two-dimensional metal-organic framework

**DOI:** 10.1038/s42004-023-00869-7

**Published:** 2023-04-18

**Authors:** Xiao-Bo Wang, Bowen Xia, Cheng-Kun Lyu, Dongwook Kim, En Li, Shu-Qing Fu, Jia-Yan Chen, Pei-Nian Liu, Feng Liu, Nian Lin

**Affiliations:** 1 Department of Physics, The Hong Kong University of Science and Technology, Hong Kong SAR, China; 2 Department of Materials Science and Engineering, University of Utah, Salt Lake City, UT 84112 USA; 3 Shanghai Key Laboratory of Functional Materials Chemistry and Institute of Fine Chemicals, East China University of Science and Technology, Shanghai, China

**Keywords:** Topological insulators, Metal-organic frameworks, Two-dimensional materials

## Abstract

The experimental realization of *p*-orbital systems is desirable because *p*-orbital lattices have been proposed theoretically to host strongly correlated electrons that exhibit exotic quantum phases. Here, we synthesize a two-dimensional Fe-coordinated bimolecular metal-organic framework which constitutes a honeycomb lattice of 1,4,5,8,9,12-hexaazatriphenylene molecules and a Kagome lattice of 5,15-di(4-pyridyl)-10,20-diphenylporphyrin molecules on a Au(111) substrate. Density-functional theory calculations show that the framework features multiple well-separated spin-polarized Kagome bands, namely Dirac cone bands and Chern flat bands, near the Fermi level. Using tight-binding modelling, we reveal that these bands are originated from two effects: the low-lying molecular orbitals that exhibit *p*-orbital characteristics and the honeycomb-Kagome lattice. This study demonstrates that *p*-orbital Kagome bands can be realized in metal-organic frameworks by using molecules with molecular orbitals of *p*-orbital like symmetry.

## Abstract (web summary)

The experimental realization of *p*-orbital systems with exotic quantum phases is desirable for the obtainment of strongly correlated materials. Here, two sublattices composed of molecules with *p*-orbital characteristics are combined to realize a *p*-orbital honeycomb-Kagome lattice in a two dimensional metal–organic framework on a Au(111) substrate.

## Introduction

The orbital degree of freedom plays a central role in condensed matter physics. Orbital design in different lattice symmetries may lead to exotic quantum phases^1–6^. Notably, as the counterpart of *p*_*z*_-orbital graphene, the *p*_*x*_/*p*_*y*_ honeycomb or other lattices can exhibit richer orbital-derived physics due to orbital degeneracy and spatial anisotropy of *p*_*x*_/*p*_*y*_ orbitals^7–20^. The *p*-orbital lattices have been proposed theoretically to host strongly correlated phases, such as Wigner-crystal-like states^7^, itinerant flat band ferromagnetism^15^, non-trivial topological phases^16^, and Chern flat band, which holds the potential to realize high-temperature fractional quantum Hall states^17–20^. The experimental realization of *p*-orbital systems with exotic quantum phases has been demonstrated in optic lattice^21,22^, and lattices of coupled micropillars^23,24^. Another approach is to use a scanning tunneling microscope to manipulate CO molecules on an atomic clean surface to create artificial lattices^25–29^. Here, we demonstrate that a *p*-orbital honeycomb-Kagome lattice (HKL) model is materialized in a magnetic 2D metal-organic framework (2D MOF) system^3,6^.

2D MOFs are predicted to be a versatile platform for realizing exotic quantum phases, including the topological insulator^30–32^, Quantum anomalous Hall effect^33^, topological Chern insulator^18^, and superconductors^34^. The interplay of diffusive *s* and *p* electrons of molecular linkers with metal *d*-orbitals coupled with specific lattice symmetries provides rich opportunities to explore orbital design. 2D MOFs with various topologies, such as Kagome and honeycomb, have been synthesized, and their band structures are mostly constructed with molecular orbitals (MOs) with *s*-orbital nature. Experimental realization of 2D MOFs comprised of two sublattices made of two types of molecules to form a honeycomb-Kagome lattice has not been reported. In this work, we synthesize a 2D MOF using two types of molecular building blocks, 5, 15-di(4-pyridyl)-10, 20-diphenylporphyrin (DPyP) and 1,4,5,8,9,12-hexaazatriphenylene (HAT), coordinated with Fe atoms on a Au (111) substrate. As resolved using scanning tunneling microscopy (STM) and scanning tunneling spectroscopy (STS), this structure constitutes a honeycomb lattice of HAT and a Kagome lattice of Fe-metalated DPyP, interconnected via Fe-N coordination. We use density-functional theory (DFT) to analyze this structure and find a series of spin-polarized Dirac-cone bands and Chern flat bands near the Fermi level. The DFT-calculated band structure can be perfectly reproduced using a tight-binding (TB) *p*-orbitals model owing to the *p*-orbital characteristics of the low-lying molecular orbitals of DPyP and HAT. This work demonstrates that by choosing molecules with orbitals of *p*-orbital characteristics, one can use 2D MOFs as platforms to study *p*-orbital physics in myriad lattices. Considering the richness of molecular orbitals in terms of symmetry, parity, and geometry, this work opens new possibilities for designing band structures in 2D MOFs.

## Results and discussion

### Structural and electronic characteristics resolved by using STM and STS

**Fig. 1:**
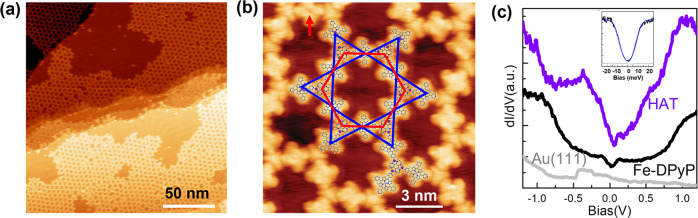
Structural and electronic properties of the HKL MOF. **a** An overview STM image of monolayer ${({{{{{\rm{HAT}}}}}}-{{{{{{\rm{Fe}}}}}}}_{3})}_{2}{({{{{{\rm{Fe}}}}}}-{{{{{\rm{DPyP}}}}}})}_{3}$ coordination network grown on Au (111) substrate. **b** A high-resolution STM showing the network structure with molecular models and a honeycomb-Kagome lattice overlaid. C, N, H, and Fe atoms are gray, blue, white, and purple, respectively. **c** Site-specific tunneling spectra acquired at Fe-DPyP and HAT. Insert: a tunneling spectrum acquired at the center of Fe-DPyP, the gray spectrum is acquired at the center of a metal-free DPyP.

After depositing DPyP and HAT molecules on an atomic flat and clean Au (111) surface held at room temperature under ultra-high vacuum conditions, two types of molecules segregate and form homo-molecular assemblies (Fig. S3). After dosing small amounts of Fe on this sample and a thermal annealing treatment at 170 °C, a porous 2D framework is formed on the surface, as shown in Fig. 1a. The framework extends over entire terraces of the Au(111) surface. Structural defects, such as domain boundaries and five-member, seven-member, and eight-member polygons (Fig. S4), are present in the network, presumably due to the flexible coordination interaction. Figure 1b is a high-resolution STM image of the hexagonal network. Two types of species are present in this structure: four-lobe squares and star-shaped triangles. In reference to the molecular appearance of DPyP and HAT shown in Fig. S3, we assign the squares to DPyP molecules and the triangles to HAT molecules. Each HAT molecule connects with three DPyP molecules and each DPyP molecule links to two HAT molecules via Fe atoms. A structural model is overlaid in Fig. 1b. In this structure, HAT molecules form a honeycomb lattice and DPyP molecules form a Kagome lattice, as represented with the red hexagonal and the blue David star, respectively, in Fig. 1b. The pore-to-pore distance is 4.61 ± 0.2 nm. Although Fe atoms are not resolved in the STM image, we propose the HAT-DPyP connection is established through a threefold Fe-N bonding motif in which a Fe atom coordinates with the pyridyl (py) ligand of DPyP and the bipyridine (bipy) ligand of HAT. A careful observation of Fig. 1b reveals that the majority of DPyP molecules feature a shallow valley in the middle of the saddle-shaped square, while the molecule pointed with the red arrow has a dip at the molecular center. This difference is attributed to the Fe metalation of central tetrapyrrole macrocycles of DPyP^35,36^, denoted as Fe-DPyP thereafter. The tunneling spectra recorded at the center of Fe-DPyP exhibit a symmetric U-shape around the zero bias with two steps at ±8.4 meV, as shown in the insert in Fig. 1c, whereas the metal-free DPyP molecules do not exhibit this feature (gray spectrum in the inset in Fig. 1c). This U-shape feature is in accordance with the spin-flip excitation of Fe^37–39^, indicating that the Fe atoms carry a magnetic moment. We term this structure as (HAT-Fe_3_)_2_ (Fe-DPyP)_3_.

Figure 1c shows the site-specific tunneling spectra acquired at HAT and Fe-DPyP (the light gray spectrum is acquired at the bare Au (111) surface). Spectroscopically, HAT shows two step-like features below the Fermi level at −1.0 and −0.3 V, two shoulders at 0.1 and 0.5 V, and a broad peak at 1.0 V. Fe-DPyP shows two shoulders at −0.8 and 1.0 V and a dip at the Fermi level, which manifests the spin-flip signature of Fe. The metal-free DPyP features a salient peak at −0.5 V and a shoulder at 1.3 V (Fig. S5).

### DFT+U calculated band structures and local density states

**Fig. 2:**
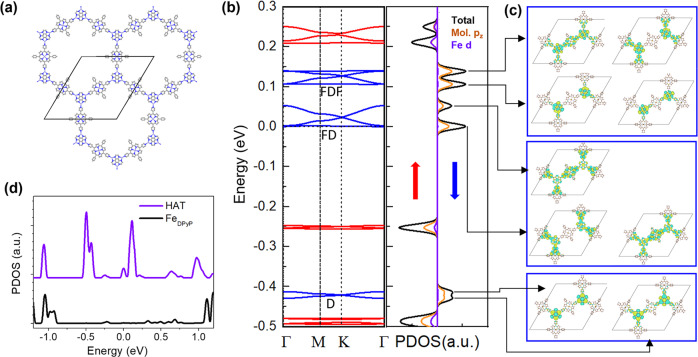
DFT analysis of the ${({{{{{\rm{HAT}}}}}}-{{{{{{\rm{Fe}}}}}}}_{3})}_{2}{({{{{{\rm{Fe}}}}}}-{{{{{\rm{DPyP}}}}}})}_{3}$ HKL MOF. **a** DFT-optimized free-standing structure of ${({{{{{\rm{HAT}}}}}}-{{{{{{\rm{Fe}}}}}}}_{3})}_{2}{({{{{{\rm{Fe}}}}}}-{{{{{\rm{DPyP}}}}}})}_{3}$. **b** Left: DFT-calculated spin-polarized band structure. Blue and red lines represent spin-down and spin-up channels. Right: spin-polarized DOS projected at Fe *d-*orbitals and molecular *p*_z_ orbitals. **c** Gamma point wavefunctions at the band edges as indicated by the arrow lines. **d** DFT-calculated DOS projected at HAT and metalation Fe in Fe-DPyP.

We perform DFT calculations with the U effect on Fe *d*-orbitals to analyze a free-standing structure of ${({{{{{\rm{HAT}}}}}}-{{{{{{\rm{Fe}}}}}}}_{3})}_{2}{({{{{{\rm{Fe}}}}}}-{{{{{\rm{DPyP}}}}}})}_{3}$. The relaxed structure is shown in Fig. 2a. The lattice constant of the optimized structure is 47 Å, which is in good agreement with the experimental data. In the threefold Fe-N coordination, the Fe-N(py) bond and the Fe-N(bipy) bonds are of the same bond length of 1.96 Å. The metalated Fe atom is bonded with four N atoms in the tetrapyrrole macrocycle core with a Fe-N bond length of 2.07 Å. The metalated Fe has a magnetic moment of 3.67 μ_B_ and the threefold coordinated Fe has a magnetic moment of 3.50 μ_B_. We analyze several magnetic configurations, including colinear ferromagnetic state and ferrimagnetic state, as well as with spin–orbit coupling (SOC). All these cases exhibit similar band structures near the Fermi level (Fig. S6). Figure 2b shows the spin-polarized bands and project-density of states (PDOS) of ${({{{{{\rm{HAT}}}}}}-{{{{{{\rm{Fe}}}}}}}_{3})}_{2}{({{{{{\rm{Fe}}}}}}-{{{{{\rm{DPyP}}}}}})}_{3}$ with a ferromagnetic ground state. There are six sets of well-separated spin-polarized bands in the energy range from −0.5 eV  to 0.3 eV. The PDOS shows that the Fe *d-* orbitals hybridize with the molecular orbitals. Thus, we argue that the Fe *d* electrons interact with the itinerant charges in the framework and lift the spin degeneracy of the electronic bands. In this work, we focus on the three sets of spin-down bands (colored in blue in Fig. 2b): a Dirac cone (denoted as D bands) around 0.4 eV below the Fermi level, a Dirac cone with one flat band (denoted as FD bands) near the Fermi level, and a Dirac cone sandwiched between two flat bands (denoted as FDF bands) around 0.1 eV above the Fermi level. Figure 2c shows the Gamma point wavefunctions at the band edges. The D bands are localized at HAT, the FD bands are at HAT and Fe-DPyP, and the FDF bands are at HAT at the low band edge and at HAT and Fe-DPyP at the high band edge, respectively.

### Using a *p*-orbital tight-binding (TB) model to reconstruct the DFT-calculated band structure

To compare with the experimentally resolved local density of states (DOS) shown in Fig. 1c, we plot the DOS projected on HAT and the metalation Fe in Fe-DPyP in Fig. 2d. Both the metalated Fe (black curve) and HAT (purple curve) feature two sets of peaks at 1.1 and −1.0 eV. The two species differ in the low energy range: the metalation Fe shows a very weak PDOS signal, whereas HAT has two sets of pronounced peaks around −0.4 and 0.3 eV. Qualitatively, the PDOS captures the characteristics of the local DOS resolved in the site-specific STS (Fig. 1c) and STS maps (Fig. S7). The difference between the PDOS and the STS can be attributed to two effects: (1) the STS signal contains the contribution of the substrate states and (2) the framework is electronically coupled with the substrate, which broadens and shifts the intrinsic DOS of the free-standing framework.

**Fig. 3:**
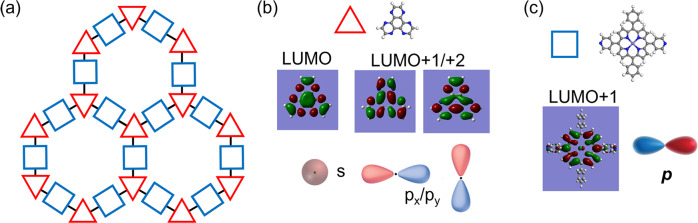
Frontier molecular orbitals of HAT and Fe-DPyP that exhibit *p*-orbital characteristics. **a** The super-atom model of ${({{{{{\rm{HAT}}}}}}-{{{{{{\rm{Fe}}}}}}}_{3})}_{2}{({{{{{\rm{Fe-DPyP}}}}}})}_{3}$, where triangle/square represents HAT/Fe-DPyP. **b** Lowest unoccupied molecular orbital (LUMO), LUMO + 1/+2 of HAT. **c** LUMO + 1 of Fe-DPyP.

As illustrated in Fig. 3a, we consider HAT and Fe-DPyP molecules as super-atoms represented by triangles and squares, respectively. Figure 3b shows the low-lying unoccupied molecular orbitals of HAT (LUMO and degenerated LUMO +1/+2) calculated using a Gaussian package. Figure 3c shows the LUMO +1 of Fe-DPyP. (Note: Fe-DPyP LUMO is localized at the central Fe atom, thus not being considered). One can find that the HAT LUMO features threefold rotation symmetry, and the degenerated LUMO + 1 and LUMO + 2 exhibit anti-phase symmetry along the vertical and horizontal central axis, respectively. The LUMO + 1 orbital of Fe-DPyP exhibits an anti-phase mirror symmetry along the central vertical axis. According to the symmetry and parity of these orbitals, we designate HAT LUMO as an *s*-orbital of the HAT super-atom (HAT-*s*), HAT LUMO + 1/+2 as HAT super-atom *p*_*x*_ and *p*_*y*_ orbitals (HAT-*p*_*x*_*/p*_*y*_), and Fe-DPyP LUMO + 1 as a rotated *p*-orbital of the DPyP super-atom (DPyP-*p*)^40^, as represented in Fig. 3b, c, respectively.

**Fig. 4:**
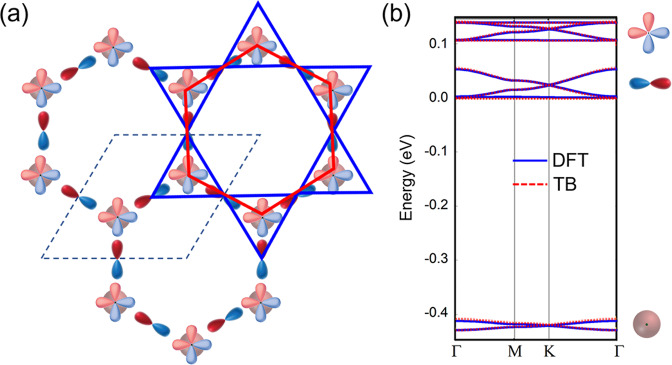
A *p*-orbital tight-binding model of the HKL lattice. **a** The HKL with *p*-orbital on Kagome lattice (in blue) and s, *p*_*x*_, *p*_*y*_ orbitals on honeycomb lattice (in red). **b** TB and DFT band structures. From the bottom up: the bands are constructed with HAT-*s*, Fe-DPyP-*p*, and HAT-*p*_*x*_/*p*_*y*_ basis, respectively.

Based on the analysis above, we construct a TB model of nine superatomic orbitals, as shown in Fig. 4a, to reproduce the DFT band structure. The TB Hamiltonian is: $H={\sum }_{i}{{\epsilon }}_{i}{c}_{i}^{+}{c}_{i}+{\sum }_{\langle i,j\rangle =\langle Hs,Dp\rangle }{t}_{sp\sigma }{c}_{i}^{+}{c}_{j}+{\sum } _{\langle i,j\rangle =\langle Dp,Hp\in \{H{p}_{x},H{p}_{y}\}\rangle }{t}_{pp\sigma }{c}_{i}^{+}{c}_{j}$ . We only consider the hoppings between nearest neighbors: where *<i,j>* represent the nearest-neighbor pair, and *Hs, Dp*, and *Hp* represent the HAT-*s*, Fe-DPyP-*p*, and HAT-*p*_*x*_/*p*_*y*_ orbitals, respectively.

Setting the hopping terms *t*_*spσ*_ = −0.04 eV and *t*_*ppσ*_ = −0.035 eV, the on-site energies ${{\epsilon }}_{{{{{{\rm{Dp}}}}}}}$ = 0.035 eV, ${{\epsilon }}_{{{{{{\rm{Hs}}}}}}}$ = −0.41 eV, ${{\epsilon }}_{{{{{{\rm{Hp}}}}}}}$ = 0.108 eV, the TB bands (dotted lines) are shown in Fig. 4b. From the bottom up, the D bands are contributed from the HAT-*s* orbital of the honeycomb lattice, the FD bands are contributed from the Fe-DPyP-*p* orbital of the Kagome lattice, and the FDF bands are contributed from the HAT-*p*_*x*_/*p*_*y*_ orbitals of the honeycomb lattice. The TB-model bands (dashed lines) are overlaid on the DFT-calculated spin-down bands (solid lines) in Fig. 4b, which show perfect agreement between the two. Therefore, we demonstrate that the band structure near the Fermi energy of this MOF can be understood as an HKL with rotated *p*-MOs. In real materials, rotating the atomic orbitals is difficult to realize. Here we show that rotating orbitals can be effectively achieved in a MOF when the molecular orbitals exhibit *p-*orbital symmetry and parity. In this context, our work provides a design principle for obtaining Dirac and flat band structures in MOFs by choosing specific molecular orbitals.

## Conclusion

In conclusion, the spin-down bands of the 2D MOF ${({{{{{\rm{HAT}}}}}}-{{{{{{\rm{Fe}}}}}}}_{3})}_{2}{({{{{{\rm{Fe}}}}}}-{{{{{\rm{DPyP}}}}}})}_{3}$ can be perfectly reconstructed using a *p*-orbital HKL TB model thank to that HAT and Fe-DPyP possess frontier molecular orbitals exhibiting *p*-orbital symmetry. This sheds light on a promising strategy of using spatial symmetry of molecular orbitals to design *p*-orbital lattices in 2D MOFs. The fully-spin-polarized band structure makes this system an appealing playground for studying Chern-bands-related quantum phenomena with broken time-reversal symmetry. More interestingly, a Chern flat band lies at the Fermi level, which provides opportunities for exploring both topological and strong correlated physics. Considering the topological bands of this structure are screened due to electronic coupling with the Au(111) substrate, future work is to synthesize this structure on an insulating substrate or an inert substrate which interacts weakly with the framework.

## Methods

### Sample preparation and measurement

All the experiments are performed in an ultra-high vacuum (UHV) system (omicron Nanotechnology) with a base pressure below 2.3 × 10^−10^ mbar. A single crystal Au (111) substrate is cleaned by Ar^+^ sputtering and annealing at 620 °C with several cycles. 5,15-di(4-pyridyl)-10,20-diphenylporphyrin (DPyP) is synthesized following the method described in ref. ^41^. 1,4,5,8,9,12-hexaazatriphenylene (HAT) is synthesized following the method described in SI (Figs. S1, S2). DPyP molecules are thermally evaporated by a molecular beam evaporator and deposited on the Au (111) substrate held at room temperature. After that, HAT molecules are deposited on the Au (111) surface using the same method. The evaporation temperatures for molecules DPyP and HAT are 330 and 165 °C, respectively. Finally, the Fe atoms are deposited on the surface using an e-beam evaporator, and the sample is annealed to 170 °C for 15 min. Without Fe, annealing at 170 °C desorbs HAT, while the remaining DPyP form close-packed molecular island. The STM measurement is performed at 4.8 K, and the bias voltage is applied to the sample plate. The standard lock-in technique with modulation of 5 mV (rms) and corresponding frequency 1.17 kHz is applied in differential tunneling spectra dI/dV measurement. The STS mapping is employed to image the electronic DOS distribution with the specific energies at different positions of the coordination network.

### DFT calculations

The first-principles calculations are carried out using the Vienna ab initio simulation package (VASP)^42^. The Perdew–Burke–Ernzerhof (PBE) functional within generalized gradient approximation (GGA)^43–45^ framework is adopted to describe the exchange-correlation functionals. The interactions between electrons and ions are treated using the projector augmented wave (PAW) method^46^ with a plane-wave cutoff energy of 450 eV. The Brillouin zone is sampled with the Gamma-only point due to the large size of the unicell. A 20 Å vacuum layer is added to decouple the interactions between neighboring layers. All atoms are relaxed until the forces are smaller than 0.01 eV/Å. Due to the correlation effect of 3*d* electrons in Fe atoms, we employed the GGA+U scheme^47^. We calculate the energy of FM and FiM states under various U values, as shown in Table. S1. The magnetic ground states are FM for U = 2–5 eV, while FiM for U = 1 eV. As shown in the density of states under different U values in Fig. S8, variation of the U value only shifts the states/bands that are contributed from Fe *d*-orbitals and does not affect the states/bands that are mainly contributed from the molecules. Also, as shown in Fig. S6, the band structures of FM and FiM states share similar features near Fermi energy. Hence, we argue that the Kagome band structure is robust under a wide-range U value.

## Supplementary information

supplementary information

Description of Additional Supplementary Files

Supplementary Data 1

## Data Availability

The data that support the findings of this study are available from the corresponding authors upon reasonable request.
