# Supplementary material for: A p-orbital honeycomb-Kagome lattice realized in a two-dimensional metal-organic framework
Source: Commun Chem. 2023 Apr 18;6:73. doi: 10.1038/s42004-023-00869-7 (PMC10113257; doi:10.1038/s42004-023-00869-7)
Supplement: Supplementary file 1 — supplementary information [file 42004_2023_869_MOESM1_ESM.pdf]

Supporting information to

A *p*-orbital honeycomb-Kagome lattice realized in a two-dimensional metal-organic framework

Xiao-Bo Wang[1], Bo-Wen Xia[2], Cheng-Kun Lyu[1], Dongwook Kim[2], En Li[1], Shu-Qing Fu[3], Jia-Yan Chen[3], Pei-Nian Liu[*3], Feng Liu[*2], and Nian Lin*[1]

[1]Department of Physics, The Hong Kong University of Science and Technology, Hong Kong SAR, China.

[2] Department of Materials Science and Engineering, University of Utah, Salt Lake City, Utah 84112, USA.

[3] Shanghai Key Laboratory of Functional Materials Chemistry and Institute of Fine Chemicals, East China University of Science and Technology, Shanghai, China.

Description of Additional Supplementary Files

Name: Supplementary Figure S1

Description: 1H NMR of HAT.

Name: Supplementary Figure S2

Description: 13C NMR of HAT.

Name: Supplementary Figure S3

Description: DPyP and HAT molecules co-exist on substrate.

Name: Supplementary Figure S4

Description: DPyP and HAT form five-member, seven-member and eight-member polygons beside the regular hexagons.

Name: Supplementary Figure S5

Description: Spatial-resolved tunnelling spectra acquired along an edge of the HKL framework where the DPyP molecule is not metalated.

Name: Supplementary Figure S6

Description: DFT-calculated band structures of a free-standing HKL framework.

Name: Supplementary Figure S7

Description: STS spectroscopy maps acquired at the indicated energies.

Name: Supplementary Table S1

Description: The total energy of FM and FiM states under different U values.

Name: Supplementary Figure S8

Description: Density of states under different U values.

Supplementary Methods

Synthesis of 1,4,5,8,9,12-hexaazatriphenylene (HAT)

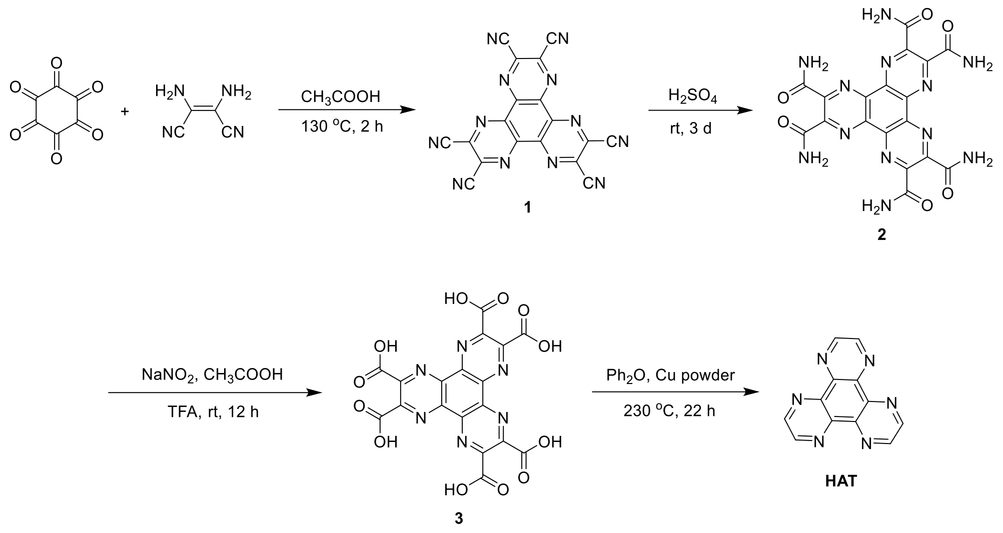

Synthesis of hexaazatriphenylenehexacarbonitrile (1). Hexaketocyclohexane octahydrate 97 (1.0 g, 6.0 mmol) and diaminomaleonitrile (2.6 g, 24.0 mmol) were added into a two-necked round-bottom flask (250 mL) equipped with magnetic stirrer and condenser and contained acetic acid (100 mL). The mixture was then heated at 130 °C for 2 h under nitrogen atmosphere. Afterwards, the resulting black mixture filtered off. The precipitate was collected, washed with hot acetic acid (3 × 50 mL), water (3 × 50 mL), ethanol (3 × 50 mL), and then dried to produce compound **1** as black powder (400 mg, 1.0 mmol, 17%). ^{13}C NMR (DMSO-d_6, 125 MHz): δ 142.0, 135.8, 114.6; HRMS (EI, TOF): calc. for $[M]^+$: 384.0369, found for $[M]^+$: 384.0370. Melting point: > 350 °C.

Synthesis of hexaazatriphenylenehexacarboxamide (2). Compound **1** (500 mg, 1.3 mmol) was dissolved in concentrated sulfuric acid (50 mL), the solution was stirred at room temperature for 72 h. Then, the reaction mixture was poured into a mixture of ice and water until a bulk precipitate formed. The precipitate was filtered off, washed with water and dried to give compound **2** (150 mg, 23%) as brown solid. ^{13}C NMR (DMSO-d_6, 125 MHz): δ 166.1, 148.2, 140.4; Melting point: > 350 °C.

Synthesis of hexaazatriphenylenehexacarboxylic acid (3). Compound **2** (500 mg, 1.0 mmol) and trifluoroacetic acid (TFA, 15.0 mL, 0.2 mol) were added into a round-bottom flask (50 mL) equipped with magnetic stirrer, then the reaction mixture was stirred at room temperature for 15 min. $NaNO_2$ (700 mg, 10.1 mmol) was added to this solution portionwise over a period of 15 min, with the temperature kept under 25 °C by cooling with an ice bath. Acetic acid (15 mL) was added, the mixture was stirred at room temperature for 12 h and poured into ice water. The precipitate was collected to produce crude product. The crude product was dissolved in $NaHCO_3$ solution (1 mol L^{-1}, 50 mL), and filtered to remove any

insoluble solid. The filtrate was treated with activated charcoal, heated to boiling, and filtered to give a clear solution that was treated with a cold NaOH solution (1 mol L^{-1}, 50 mL). An immediate precipitation occurred, and complete precipitation was effected by the addition of ethanol (50 mL). The product was filtered, washed with 10% HCl solution (3 × 50 mL) and water (3 × 50 mL), and dried under vacuum to afford compound **3** (62 mg, 12%) as yellow solid. ^{13}C NMR (DMSO-d_6, 125 MHz): δ 165.7, 146.5, 141.7; Melting point: > 350 °C.

Synthesis of 1,4,5,8,9,12-Hexaazatriphenylene (HAT). A mixture of compound **3** (200 mg, 0.41 mmol), Cu powder (500 mg, 7.9 mmol), and freshly distilled diphenyl ether (5 mL) is stirred at 230 °C for 22 h under a dry nitrogen atmosphere. The reaction is then cooled and filtered, and the solid is washed with petroleum ether (3 × 50 mL) to remove adsorbed diphenyl ether. The crude solid was purified by a short column of neutral alumina and eluted with $CHCl_3$ to afford **HAT** (20 mg, 21%) as white solid. ^{1}H NMR ($CDCl_3$, 400 MHz): δ 9.3 (s, 6H); ^{13}C NMR ($CDCl_3$, 125 MHz): δ 147.0, 142.3; HRMS (ESI, TOF): calc. for $[M+H]^+$: 234.0654, found for $[M+H]^+$: 235.0734. Melting point: > 350 °C.

SUPPORTING DATA

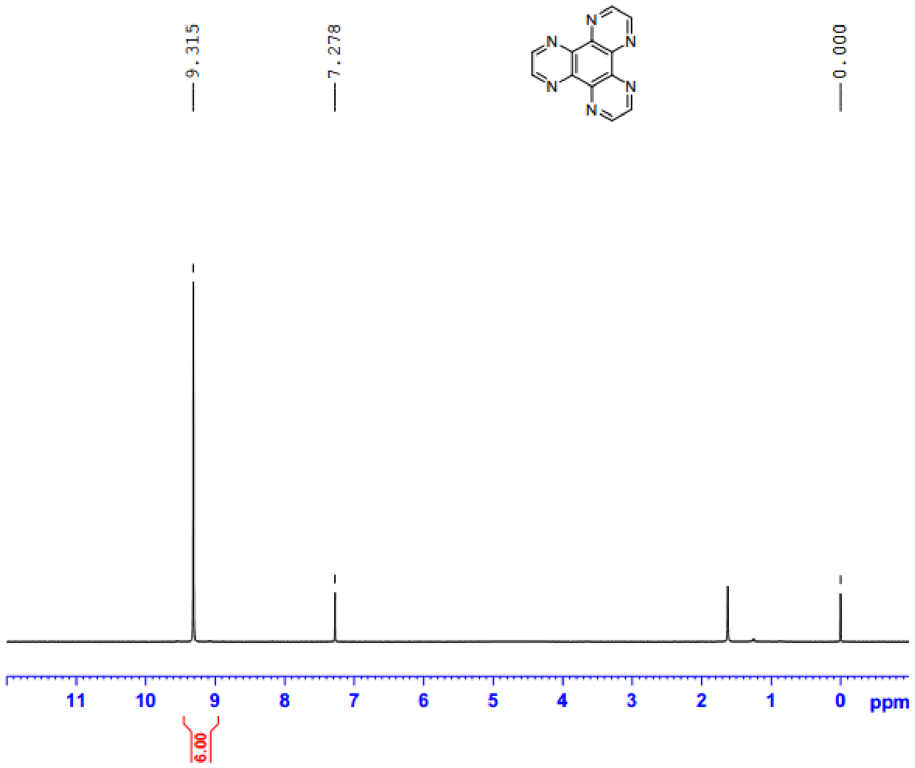

Figure S1. ^{1}H NMR of HAT.

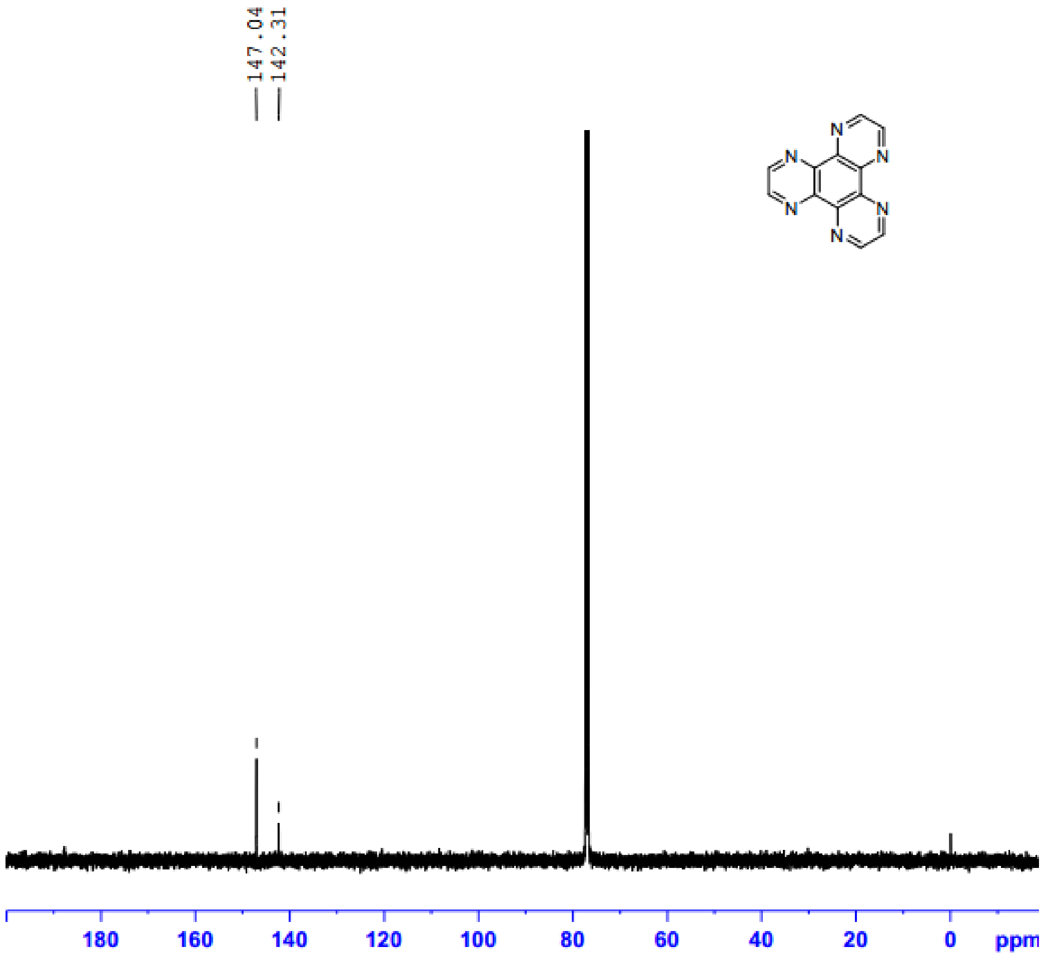

Figure S2. **^{13}C NMR of HAT.**

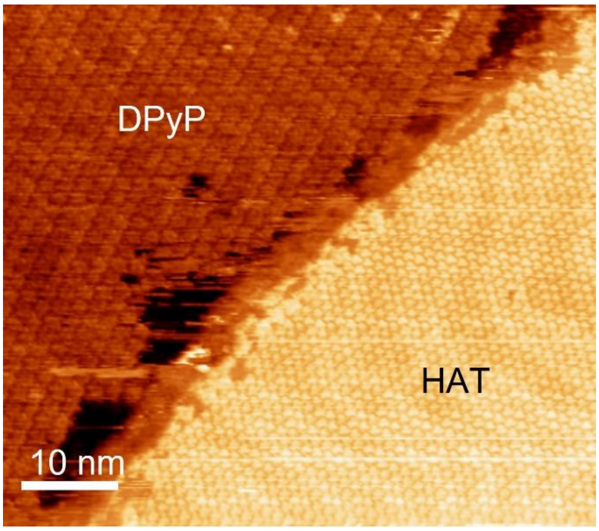

Figure S3. DPyP and HAT molecules co-exist on substrate.

With sequential deposition of DPyP and HAT molecules on the Au (111) surface, two types of molecules segregate and assemble as homo-molecular domains on the surface.

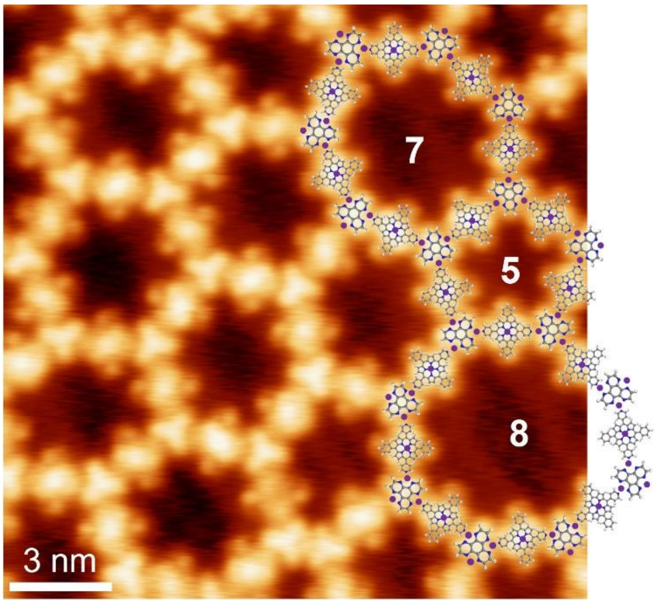

Figure S4. DPyP and HAT form five-member, seven-member and eight-member polygons beside the regular hexagons. Such structural variations indicate that the coordination bonds that connect DPyP and HAT are flexible in bond angle.

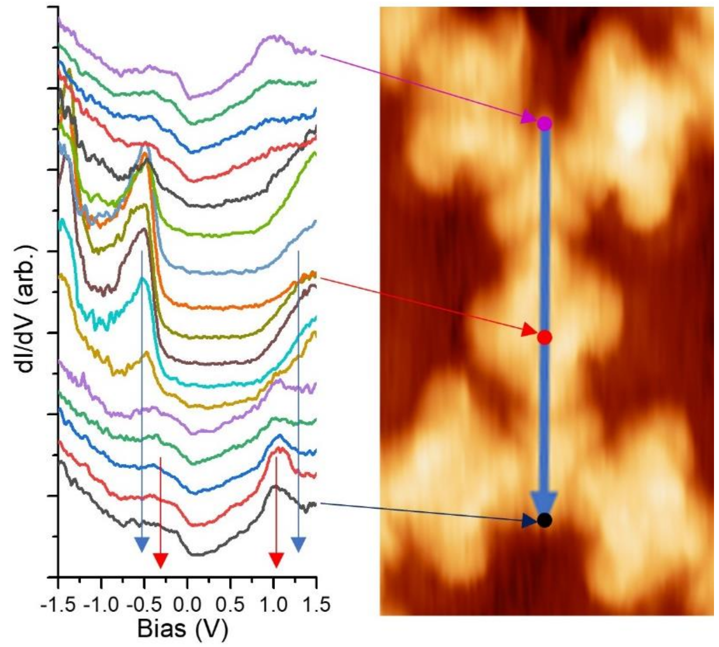

Figure S5. Spatial-resolved tunnelling spectra (left panel) acquired along an edge of the HKL framework where the DPyP molecule is not metalated (right panel).

The DPyP shows a peak at -0.5 and a shoulder at 1.3 V (blue arrows). Two HAT show a shoulder at -0.3 V and a peak at 1.0 V (red arrows).

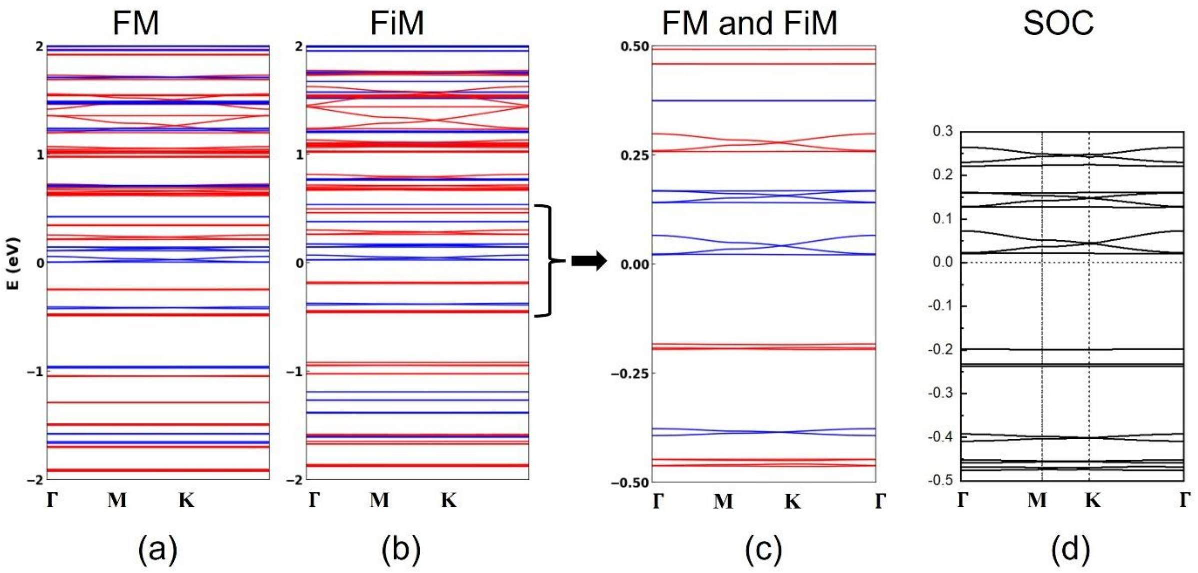

Figure S6. DFT-calculated band structures of free-standing HKL framework. (a) and (b) Colinear spin-polarized ferromagnetic (FM) ground state and ferrimagnetic (FiM) ground state. (c) Enlarged view of (a) and (b) near the Fermi level. (d) With spin-orbit coupling (SOC).

The band structures near the Fermi level from -0.5 eV to 0.5 eV of the three configurations are almost overlapped.

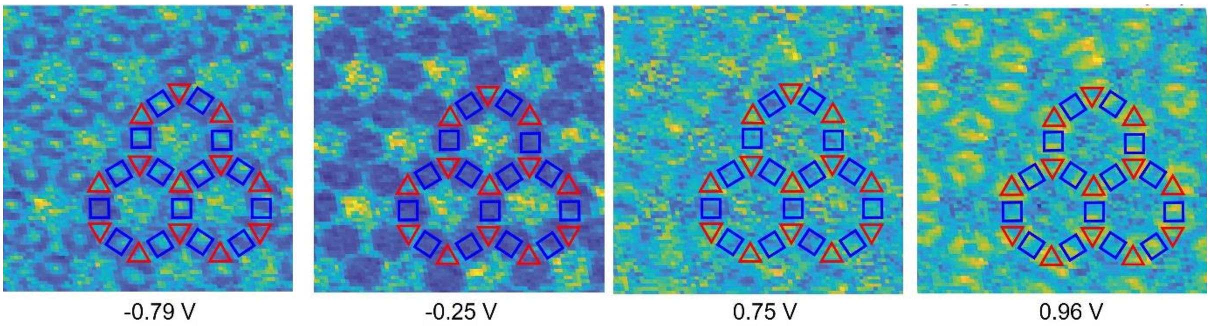

Figure S7. STS spectroscopy maps acquired at the indicated energies. HAT and DPyP are represented with red triangles and blue squares, respectively,

Both molecules are visible at -0.79 V and 0.96 V, which corroborates the DOS features at -1.0 eV and 1.0 eV in Fig. 2d; HAT is more pronounced at -0.25 V and 0.75 V, which corroborates the DOS features at -0.5 eV and 0.2 eV in Fig. 2d.

Table S1. The total energy of FM and FiM states under different U values.

	$E_{U=1eV}$ (eV)	$E_{U=2eV}$ (eV)	$E_{U=3eV}$ (eV)	$E_{U=4eV}$ (eV)	$E_{U=5eV}$ (eV)
FM	-1982.9673	-1979.3403	-1976.1921	-1973.4698	-1971.1362
FiM	-1983.2708	-1979.1280	-1975.3319	-1972.0678	-1969.1971

The energy of FM and FiM states under different U values are shown in the Table. S1. The magnetic ground states are FM for U=2~5 eV, while being FiM for U=1eV.

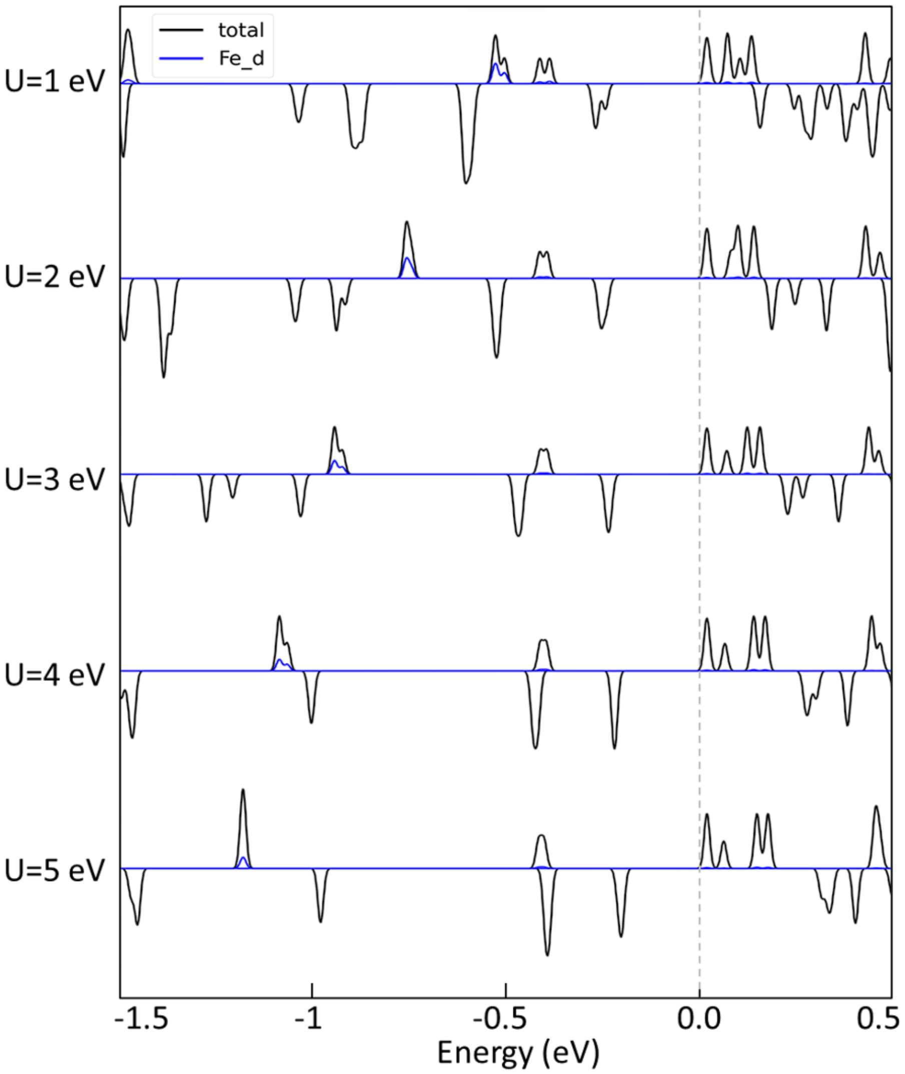

Figure S8 Density of states under different U values.

Variation of U value shifts the states/bands that are contributed from Fe d orbitals and does not affect the states/bands that are mainly contributed from molecule.
