# Supplementary material for: A p-orbital honeycomb-Kagome lattice realized in a two-dimensional metal-organic framework
Source: Commun Chem. 2023 Apr 18;6:73. doi: 10.1038/s42004-023-00869-7 (PMC10113257; doi:10.1038/s42004-023-00869-7)
Supplement: Supplementary file 2 — Description of Additional Supplementary Files [file 42004_2023_869_MOESM2_ESM.pdf]

Description of Additional Supplementary Files

File name: Supplementary Data 1

Description: Atomic coordinates of the DFT calculated HKL structure.
